# Health-Related Quality of Life and Work-Related Outcomes for Patients With Mild-to-Moderate Ulcerative Colitis and Remission Status Following Short-Term and Long-Term Treatment With Multimatrix Mesalamine: A Prospective, Open-Label Study

**DOI:** 10.1093/ibd/izx041

**Published:** 2018-01-18

**Authors:** Aaron Yarlas, Geert D’Haens, Mary Kaye Willian, Megan Teynor

**Affiliations:** 1 Optum, Johnston, RI, United States; 2 Inflammatory Bowel Disease Centre, Academic Medical Centre, University of Amsterdam, The Netherlands; 3 Shire, Wayne, PA, United States; 4 Shire, Lexington, MA, United States

**Keywords:** multimatrix mesalamine, ulcerative colitis, health-related quality of life, work-related outcomes, presenteeism

## Abstract

**Background:**

Disease activity of patients with ulcerative colitis (UC) predicts health-related quality of life (HRQL) and work-related outcomes (eg, absenteeism, productivity). We tested whether outcomes differed among patients in complete (clinical and endoscopic) remission, partial remission, or not in remission following treatment with multimatrix mesalamine.

**Methods:**

Data were from an open-label, multicountry, prospective trial (ClinicalTrials.gov identifier: NCT01124149) of 717 adults with active mild-to-moderate UC treated with 4.8 g/day multimatrix mesalamine tablets for 8 weeks (induction period); 459 patients who achieved partial or complete remission received daily 2.4 g/day multimatrix mesalamine for 12 additional months (maintenance period). HRQL (SF-12v2 Health Survey and Short Inflammatory Bowel Disease Questionnaire) and work-related outcomes (Work Productivity and Activity Impairment questionnaire) were assessed at baseline and final visits of each treatment period. Differences in scores by remission status within each treatment period were tested using analysis of variance and analysis of covariance models, whereas mixed-effects models with repeated measures tested changes over time.

**Results:**

At their final visit of each treatment period, patients in partial remission scored significantly better on all HRQL and work-related domains than patients not in remission (all Bonferroni-adjusted *P* < 0.05). Scores for patients in partial remission were, almost without exception, statistically equivalent to those for patients in complete remission. Fluctuating between complete and partial remission during maintenance treatment had no impact on outcomes.

**Conclusions:**

Patients in partial remission following multimatrix mesalamine treatment had HRQL and work-related outcomes equivalent to patients in complete remission. Achievement and maintenance of partial remission may be sufficient for improvements in patients’ functioning, well-being, and work performance.

Ulcerative colitis (UC) is an inflammatory bowel disease (IBD) that causes chronic inflammation and ulceration of the colon. UC follows a relapse-remittent course, with recurrent flares accompanied by clinical symptoms that include abdominal pain, rectal bleeding, increased stool frequency, and diarrhea. Because this disease is currently incurable (barring surgical removal of the colon), medical treatments for UC have focused on inducing remission in those with active disease, and then maintaining remission once it has been achieved.

Remission in UC can be assessed along multiple dimensions. Clinical remission is generally determined by the presence, frequency, and severity of symptoms such as rectal bleeding and stool frequency. Endoscopic remission is based on clinical ratings of mucosal appearance, which can include vascular pattern, granularity, ulceration, and friability. Histological remission is evaluated based on evidence including distortion of crypt architecture, crypt abscesses, and inflammation of cells in the lamina propria. However, within each of these dimensions, there has been no consensus regarding which features are necessary and sufficient for defining remission.^1,2^

This lack of consensus is highlighted by the lack of standardized measures for capturing remission within each of these dimensions. Reviews of clinical trials in UC populations have identified dozens of measures used to assess clinical, endoscopic, and/or histologic remission, with no validated, gold standard instrument identified from among them.^1–6^

Historically, research on the treatment for UC focused on the reduction of clinical symptoms. However, in the past decade, there has been growing consensus for the achievement of mucosal healing as a crucial treatment endpoint.^6–10^ This has led to the increased inclusion in clinical trials of composite measures of both clinical symptoms and mucosal health, including the UC Disease Activity Index (UC-DAI)^11^ and the Mayo Clinic Score.^12^ Patients achieving both clinical remission and normal mucosal health are considered to be in complete remission.^1,10,13^

Across studies with varying definitions and measurement of remission, what remains consistent are findings that patients whose UC is in remission demonstrate better health-related quality of life (HRQL) than patients with active disease. Better scores on disease-specific HRQL measures (eg, Inflammatory Bowel Disease Questionnaire [IBDQ]) have been observed for UC patients classified as being in clinical remission^14–23^ or endoscopic remission^18,19,24^ than for patients with active disease. Similar benefits on measures of generic HRQL (eg, SF-36^®^ Health Survey [SF-36]; EuroQol-5D [EQ-5D]) have been found for patients whose UC is in clinical remission,^19,21,22,25^ or in complete (ie, both clinical and endoscopic) remission^26^ when compared to their counterparts with active disease.

Studies examining the association between remission status and work-related outcomes (WRO), such as absenteeism, productivity, and work disability status, have also shown advantages for patients whose UC is in remission. For example, patients with UC in clinical remission are more likely to be employed, less likely to receive disability compensation, use fewer sick days, and report higher productivity when working than patients with active UC.^27,28^

The evidence cited above indicates that patients with UC who are classified as being in remission experience better HRQL and WRO than patients classified with active disease. What has not been explored, and thus remains unclear, is whether achievement of complete remission is necessary to experience benefits in these outcomes. In other words, it has not yet been established whether patients in an intermediate disease state—partial remission—have HRQL and WRO more similar to patients who are not in remission, or those in complete remission. The purpose of the analyses presented here was to assess HRQL and WRO among patients with UC who were classified into one of three remission categories—complete remission, partial remission, or not in remission—after receiving 8 weeks of induction treatment or 12 months of maintenance treatment with multimatrix mesalamine tablets (Cosmo Technologies Ltd, Wicklow, Ireland) in a multicenter, multinational, open-label trial.

## METHODS

### Study Design and Sample

Data for these analyses were from a phase 3b/4 multinational, open-label, prospective trial of multimatrix mesalamine treatment for adults (≥18 years) with UC (MOMENTUM study; ClinicalTrials.gov identifier: NCT01124149).^29^ Patients were enrolled at sites from 14 countries: Canada, the United States (US), Colombia, Belgium, Czech Republic, France, Hungary, Ireland, Poland, Romania, Spain, United Kingdom, South Africa, and India.

Trial eligibility was limited to patients diagnosed at screening with active mild-to-moderate UC. Diagnosis was determined using a modified version of the UC-DAI (_MOD_UC-DAI),^30^ and included the presence of an acute flare with a total score between 4 and 10 (inclusive), an endoscopy rating ≥1 and a physician global assessment (PGA) rating ≤2. This modification to the UC-DAI increased the stringency of endoscopic assessment by coding evidence for mucosal friability as an indicator of moderate disease (ie, endoscopy rating = 2), rather than of mild disease (endoscopy rating = 1) as in Sutherland’s original scale.^11^

The trial consisted of an 8-week induction treatment period followed by a 12-month maintenance treatment period. During the induction treatment period, patients received 4.8 g/day of multimatrix mesalamine once daily (QD). At the 8-week visit of the induction phase, patients were classified into remission status groups based on _MOD_UC-DAI component and total scores (Table 1). Patients with a _MOD_UC-DAI score ≤1, scores of 0 for both stool frequency and rectal bleeding components, and a reduction from baseline of at least 1 point on the endoscopy component, were classified as being in complete remission. Patients with a _MOD_UC-DAI score ≤3, a combined score of 0 or 1 on the stool frequency and rectal bleeding components, and who did not meet the criteria for complete remission were classified as being in partial remission. Patients with a _MOD_UC-DAI score >3, or who had a combined score on the stool frequency and rectal bleeding components >1, were classified as being not in remission. Additionally, early withdrawal patients who did not complete the full 8 weeks of induction treatment were automatically classified as being not in remission, regardless of their _MOD_UC-DAI scores at their final induction period visit.

**Table 1.** Classification of Remission Status

Remission Status	Criteria for Classification
Complete remission	_MOD_UC-DAI total score ≤1, AND Stool frequency score = 0, AND Rectal bleeding score = 0, AND ≥1-point reduction in endoscopy score from baseline
Partial remission	_MOD_UC-DAI total score ≤3, AND Combined stool frequency score and rectal bleeding ≤1, AND Not in complete remission
Not in remission	_MOD_UC-DAI total score >3, OR Combined stool frequency score and rectal bleeding >1, OR Did not complete full course of treatment (early withdrawal)

MODUC-DAI, modified version of the Ulcerative Colitis Disease Activity Index.

Patients who completed the full course of induction treatment and who achieved partial or complete remission were eligible for enrollment in the maintenance treatment period, during which they received 2.4 g/day of multimatrix mesalamine QD. Patients’ remission status at the end of the maintenance treatment period was determined using the same classification criteria described above (and in Table 1).

**FIGURE 1.**
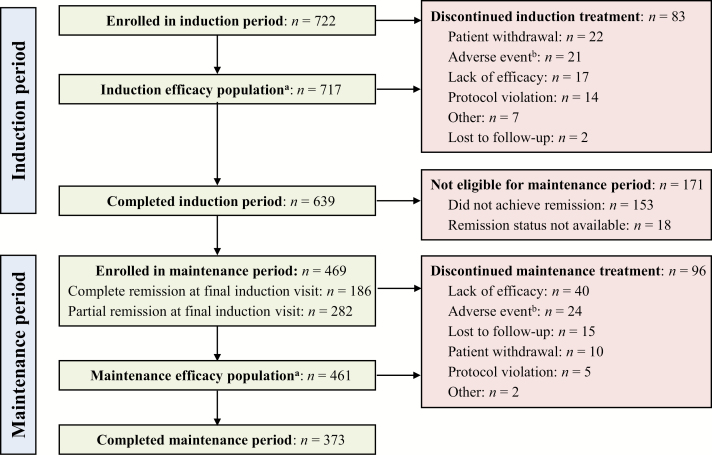
Flow Chart for Disposition of Trial Patients. ^a^The efficacy population within each period was defined as all patients who took at least 1 dose of the investigational product and had at least 1 postdose efficacy assessment during that period. ^b^The specific adverse events experienced by patients in this study have been detailed elsewhere.^29^

A flowchart of the trial design, including patient disposition for each treatment period, is presented in Figure 1. A total of 892 patients were screened; of these, 722 who met all inclusion and exclusion criteria were enrolled in the trial. The induction sample included the 717 patients who took at least 1 dose of multimatrix mesalamine and had at least 1 post-dose efficacy assessment during the induction treatment period. The final induction period visit occurred at 8 weeks for patients who completed the full course of treatment or at the early withdrawal visit for patients who did not complete treatment. Patient-reported outcomes (PRO) measures were administered at postscreening baseline, and at the final induction period visit.

A subset of 468 patients from the induction sample who had completed induction treatment and who were classified as having achieved complete or partial remission at the week 8 visit were enrolled in the maintenance treatment period. The maintenance subset included the 459 of these patients who received at least 1 dose of multimatrix mesalamine and had at least 1 postdose efficacy assessment during the maintenance treatment period. The week 8 visit of the induction period was treated as the initial (month 0) visit for the maintenance period. PRO measures also were administered at the final maintenance period visit, which occurred at 12 months for patients who completed the maintenance period or at the early withdrawal visit for patients who withdrew from the trial before 12 months. The _MOD_UC-DAI and measures of HRQL and WRO were administered at baseline, the final induction period visit, and the final maintenance period visit.

### Ethical Considerations

This trial was conducted with approval of an Institutional Review Board at each site and in accordance with current applicable regulations, International ICH Good Clinical Practice and local ethical and legal requirements, and with the principles of the 18th World Medical Assembly (Helsinki 1964) and amendments of the 29th (Tokyo 1975), 35th (Venice 1983), 41st (Hong Kong 1989),and 48th (South Africa 1996) World Medical Assemblies, Declaration of Helsinki. All participating patients provided written informed consent at screening.

### Measures of HRQL and WRO

The Short Inflammatory Bowel Disease Questionnaire (SIBDQ)^31^ was used to measure disease-specific HRQL. The SIBDQ, a 10-item, self-reported survey with a 2-week recall period, captures the impact of IBD on 4 HRQL domains: bowel symptoms (3 items capturing abdominal pain, flatulence, and urge to defecate), systemic symptoms (2 items capturing fatigue and weight maintenance), emotional function (3 items capturing depression, stress, and anger), and social function (2 items capturing frequency of canceling and being limited in social activities). A total score can be calculated from responses to all 10 items. For all domains and the total score, higher values reflect better HRQL. Based on findings from a previous study of patients with Crohn’s disease, the threshold for a minimal clinically important difference (MCID), which indicates a clinically meaningful improvement in a patient’s health, has been estimated as an increase of 9 points in the SIBDQ total score.^31^

Generic HRQL was measured using the SF-12v2^®^ Health Survey (SF-12v2), which is a self-reported survey of functional health and well-being with a 4-week recall period.^32^ The SF-12v2 captures 8 domains of HRQL: Physical Functioning (PF), Role limitations due to Physical health problems (RP), Bodily Pain (BP), General Health perception (GH), Vitality (VT), Social Functioning (SF), Role limitations due to Emotional health problems (RE), and Mental Health (MH). Physical Component Summary (PCS) and Mental Component Summary (MCS) scores, which provide global metrics of physical and mental health, respectively, are each calculated from weighted summations of all domains. SF-12v2 domain and summary scores are norm-based, having been standardized using scores from a national probability sample of 6012 noninstitutionalized adults in the US who participated in a 2009 Internet-based survey conducted by QualityMetric, Incorporated.^32^ All are expressed as T-scores, with a mean of 50 and a standard deviation of 10, and with higher values indicating better HRQL. A domain or summary score of 50 indicates equivalence to the general population mean, and thus designates a “normalized” score. MCID thresholds for clinically meaningful improvement, derived using data from both a general population sample and from multiple samples across a variety of health conditions, have been estimated as an increase of 6 points in the SF-12v2 PCS score and of 7 points in the SF-12v2 MCS score.^32^

The Work Productivity and Activity Impairment (WPAI) instrument is a 6-item, self-report survey of the impairments in WRO due to health problems over the previous 7 days.^33^ A disease-specific version of this instrument was used to capture impairment on these outcomes specific to UC-related problems (WPAI:UC). Patients’ responses to items were used to calculate the impact of UC on 4 domains: Absenteeism (work time missed), Presenteeism (impairment while working), Overall Work Impairment (overall productivity loss, accounting for both absenteeism and presenteeism), and Activity Impairment (impairment in non-work activities, such as shopping and child care). Patients who reported not being employed during the previous 7-day period were scored only on the Activity Impairment domain. Scores from all domains are expressed as percentages (0–100%) of impairment, with lower values indicating less impairment due to UC. The MCID threshold for clinically meaningful improvement in the Overall Work Impairment domain, based on data from a sample of patients with Crohn’s disease, has been estimated as a decrease of 7 points.^34^

### Statistical Analysis

Analytic models including scores from induction period visits only (ie, baseline and final induction period visits) were conducted using the induction sample, whereas models including scores from maintenance period visits only (ie, month 0 and final maintenance period visits) or across both induction and maintenance period visits were conducted using the maintenance subset.

Differences in baseline mean scores on SIBDQ, SF-12v2, and WPAI:UC domains among remission status groups were tested separately for the induction sample and for the maintenance subset, with remission status determined at the final induction phase visit for the former and at the final maintenance period visit for the latter. Univariate one-way analysis of variance (ANOVA) models, with remission status group as a between-subjects factor, were tested for each domain. Across all models within each instrument, Hommel’s stepwise method^35^ was used to adjust *P*-values of omnibus F-tests to control for inflation of the Type I error rate due to multiplicity of tests. For domains in which omnibus tests were statistically significant, post hoc tests using Bonferroni-corrected *P*-values to control for alpha inflation due to multiplicity tested for statistical significance of pairwise differences in mean scores between complete remission, partial remission, and not in remission groups.

Differences in scores on HRQL and WRO domains among remission status groups were assessed for the induction sample at the final induction period visit and for the maintenance subset at the final maintenance period visit. Univariate one-way analysis of covariance (ANCOVA) models were tested for each SIBDQ, SF-12v2, and WPAI:UC domain. In each model, the domain score at the final period visit was the dependent variable, with remission status group at that visit as a between-subjects factor, and with patient age, gender, duration of disease, disease extent (coded ordinally across 3 levels: left sided, involvement of transverse colon, and pancolitis), and the value of that domain at the initial period visit (ie, baseline visit for the induction period, month 0 visit for the maintenance period) included as a covariate. Across all models within each instrument, Hommel-adjusted *P*-values were used for omnibus tests, and pairwise remission status group differences in mean scores of domains for which omnibus tests were statistically significant were tested using Bonferroni-corrected *P*-values.

A responder analysis was used to examine whether the likelihood of a patient experiencing a clinically meaningful improvement in HRQL or WRO following induction treatment varied as a function of their posttreatment remission status. For each of the 4 outcomes for which MCID thresholds have been estimated—SIBDQ total score, SF-12v2 PCS, SF-12v2 MCS, and the Overall Work Impairment domain of the WPAI:UC—each patient in the induction sample was classified as a responder if the magnitude of their improvement in that outcome score following induction treatment exceeded the MCID thresholds previously estimated for each of these measures (ie, 9-point increase in SIBDQ total score, 6-point increase in SF-12v2 PCS score, 7-point increase in SF-12v2 MCS score, and 7-point decrease in WPAI:UC Overall Work Impairment score). For each of these outcomes, the proportion of responders was calculated for each remission status group, and Chi-square tests of association were used to assess whether this proportion statistically differed among remission status groups. Outcomes for which the omnibus Chi-square test indicated statistically significant group differences, standardized residuals were used to test for the significance of pairwise differences in the proportion of responders between complete remission, partial remission, and not in remission groups.

Repeated-measures analyses were conducted to examine whether patterns of changes in key HRQL and WRO measures (again, SIBDQ total score, SF-12v2 PCS, SF-12v2 MCS, and the Overall Work Impairment domain of the WPAI:UC) over the course of both induction and maintenance treatment periods varied as a function of patients’ remission status at their final maintenance period visit. Mixed-effects models with repeated measures (MMRM) were conducted for each of these outcomes based on scores at baseline, month 0, and final maintenance period visits for patients in the maintenance subset. In each MMRM, the patient was treated as a random effect whereas fixed effects included visits, patients’ remission status at their final maintenance period visit, and the interaction between visit and remission status, with patients’ age, gender, duration of disease, and disease extent at study baseline included as covariates. An unstructured covariance matrix was assumed for residuals. For each of the 4 models, estimated mean scores on the tested outcome were derived for each remission status group at each visit. Outcomes for which the omnibus F-tests for all fixed effects were statistically significant (post hoc tests using Bonferroni-corrected *P*-values to control for alpha inflation due to multiplicity) tested for statistically significant changes in mean scores across visits (from baseline to week 8/month 0, and from month 0 to the final maintenance period visit) within each remission status group.

Analyses also were conducted to test whether trajectory of remission status from the end of the induction period to the end of the maintenance period had any impact on HRQL and WRO for patients in the maintenance subset. If it is the case that HRQL and WRO are equivalent for patients in partial and complete remission, then we should observe no changes in outcomes over time regardless of whether patients’ remission status fluctuates between partial and complete remission or remains the same from month 0 to the final maintenance period visit. For this analysis, 4 groups of patients within the maintenance subset were defined in terms of remission status at the beginning (month 0) and the end (final visit) of the maintenance period: Complete remission → complete remission; partial remission → partial remission; complete remission → partial remission; and partial remission → complete remission. To test for differences in HRQL and WRO across these 4 possible trajectories, univariate two-factor ANCOVA models were tested for each domain of each of the 3 outcomes instruments. In each model, the domain score at the final maintenance period visit was the dependent variable, with remission status at month 0, remission status at the final maintenance period visit, and the interaction between remission status at month 0 and remission status at the final maintenance period visit as between-subjects factors, and with age, gender, disease duration, disease extent, and month 0 values as covariates. Across all models within each instrument, Hommel’s stepwise method^35^ was used to adjust *P*-values of omnibus F-tests for each effect to control for inflation of the Type I error rate due to multiplicity of tests.

Multivariable regression models were used to test the degree to which change in HRQL and WRO for the induction sample during the induction period and for the maintenance subset during the maintenance period were predicted by patient characteristics and by remission status at the final period visit. Models were conducted for each SIBDQ, SF-12v2, and WPAI:UC domain, with predictors including remission status, age, gender, duration of disease, disease extent, and the value of that domain at the initial period visit.

## RESULTS

Demographic and clinical characteristics were similar for patients in the induction sample and those in the maintenance subset (Table 2). At the end of each period, approximately 40% of patients were in partial remission, as compared to one-quarter of patients in complete remission and one-third of patients not in remission.

**Table 2.** Patient Characteristics for Induction Sample and Maintenance Subset

	Induction Sample (*n* = 717)	Maintenance Subset (*n*= 459)
**Gender, *n* (%**)		
Male	308 (43.0)	200 (43.6)
Female	409 (57.0)	259 (56.4)
**Baseline age (years), mean (SD**)	42.9 (14.0)	42.7 (14.2)
**Baseline BMI, mean (SD**)	24.4 (4.9)	24.3 (4.8)
**Race, *n* (%**)		
American Indian/Alaskan native	13 (1.8)	12 (2.6)
Asian	206 (28.7)	142 (30.9)
Black/African American	10 (1.4)	7 (1.5)
White	428 (59.7)	255 (55.6)
Other	60 (8.4)	43 (9.4)
**Baseline**_**MOD**_ **UC-DAI, mean (SD**)		
Stool frequency	1.7 (0.8)	1.6 (0.8)
Rectal bleeding severity	1.3 (0.7)	1.2 (0.7)
Mucosal appearance	1.9 (0.5)	1.9 (0.5)
Physician global assessment	1.6 (0.5)	1.6 (0.5)
Total score	6.6 (1.6)	6.3 (1.5)
**Duration of disease (months), mean (SD**)	64.1 (84.9)	64.2 (79.6)
**Newly diagnosed, *n* (%**)	70 (9.8)	38 (8.3)
**Disease extent, *n* (%**)		
Left sided	557 (77.7)	357 (77.8)
Involvement of transverse colon	50 (7.0)	33 (7.2)
Pancolitis	110 (15.3)	69 (15.0)
**Remission status at final induction period visit, *n* (%**)^a^		
Complete remission	186 (26.6)	182 (39.7)
Partial remission	282 (40.3)	277 (60.3)
Not in remission	231 (33.1)	0 (0.0)
**Remission status at final maintenance period visit, *n* (%**)^b^		
Complete remission	—	159 (39.7)
Partial remission	—	103 (25.7)
Not in remission	—	139 (34.7)

BMI, body mass index; _MOD_UC-DAI, modified version of the Ulcerative Colitis Disease Activity Index; SD, standard deviation.

a The 78 patients who did not complete induction treatment were classified as not in remission; remission status was missing for 18 patients in the induction sample.

b The 87 patients who did not complete maintenance treatment were classified as not in remission; remission status was missing for 58 patients in the maintenance subset.

Baseline scores for SIBDQ, SF-12v2, and WPAI:UC domains by remission status group within the induction sample and the maintenance subset are presented in Table 3. Within each patient sample, no statistically significant differences among remission status groups were observed for any domain.

**Table 3.** Observed Baseline SIBDQ, SF-12v2, and WPAI:UC Scores by Remission Status Groups in the Induction Sample and Maintenance Subset

Measure	Induction Sample^a^						Maintenance Subset^b^					
	Not in Remission		Partial Remission		Complete Remission		Not in Remission		Partial Remission		Complete Remission	
	N	Mean (SD)	N	Mean (SD)	N	Mean (SD)	N	Mean (SD)	N	Mean (SD)	N	Mean (SD)
**SIBDQ**												
Bowel Symptoms	184	12.2 (3.1)	204	12.8 (3.1)	141	12.8 (3.1)	108	12.6 (3.1)	76	13.0 (3.2)	109	12.7 (3.2)
Systemic Symptoms	185	8.5 (2.8)	209	9.1 (2.6)	142	9.0 (2.4)	109	9.1 (2.6)	77	8.6 (2.7)	112	9.2 (2.6)
Emotional Function	184	12.2 (3.9)	205	13.1 (3.9)	143	13.0 (3.8)	109	13.1 (3.7)	75	12.7 (4.3)	111	12.9 (3.9)
Social Function	185	8.5 (3.3)	208	9.2 (2.9)	143	9.1 (2.8)	108	9.2 (2.9)	76	9.1 (3.2)	113	8.9 (2.9)
**SF-12v2**												
Physical Functioning	204	46.1 (9.5)	246	46.5 (8.8)	155	45.8 (9.3)	127	48.0 (8.4)	90	44.8 (10.0)	129	45.5 (9.2)
Role Physical	203	42.6 (8.6)	245	44.5 (7.7)	154	44.2 (7.3)	127	45.2 (7.4)	89	44.1 (8.0)	128	43.9 (7.6)
Bodily Pain	203	42.8 (9.9)	244	44.1 (8.6)	153	43.1 (9.0)	125	44.7 (9.1)	89	43.8 (9.0)	128	42.6 (8.6)
General Health	206	42.1 (10.6)	245	41.6 (10.2)	155	40.6 (9.2)	126	41.5 (10.3)	90	41.1 (9.8)	129	40.6 (9.9)
Vitality	202	45.6 (10.4)	242	47.2 (9.4)	155	47.4 (8.1)	126	47.0 (9.2)	89	47.2 (8.8)	127	47.1 (9.1)
Social Functioning	205	40.9 (9.7)	246	42.9 (9.2)	155	43.3 (8.5)	127	43.9 (9.0)	90	43.1 (9.9)	129	42.0 (8.3)
Role Emotional	204	41.7 (10.2)	245	42.4 (9.7)	154	40.9 (8.6)	126	43.0 (9.7)	89	40.2 (10.4)	129	41.4 (8.8)
Mental Health	205	43.5 (10.4)	246	44.5 (9.2)	155	44.3 (8.5)	127	44.7 (9.0)	90	42.8 (10.4)	129	44.5 (8.6)
**WPAI:UC**												
Absenteeism	102	14.8 (27.1)	111	13.4 (24.4)	79	11.8 (19.8)	62	9.7 (21.0)	46	17.1 (24.4)	63	10.7 (20.7)
Presenteeism	104	41.7 (26.4)	116	32.4 (23.4)	80	34.0 (22.4)	63	30.2 (21.4)	47	32.3 (23.9)	65	35.4 (24.7)
Overall Work Impairment	100	47.2 (30.1)	111	38.8 (27.4)	78	40.8 (26.3)	62	36.7 (25.8)	45	42.2 (28.5)	63	38.8 (27.0)
Activity Impairment	172	46.6 (27.2)	190	38.5 (25.2)	132	39.7 (23.9)	102	37.9 (25.4)	75	37.7 (25.6)	103	39.8 (25.3)

SD, standard deviation; SF-12v2, SF-12v2 Health Survey; SIBDQ, Short Inflammatory Bowel Disease Questionnaire; WPAI:UC, Work Productivity and Activity Impairment–Ulcerative Colitis.

a Remission status based on classification at the final induction period visit.

b Remission status based on classification at the final maintenance period visit.

Within each sample, one-way analysis of variance tests found no statistically significant effects of remission status for any models (all Hommel-adjusted *P* > 0.30).

Models for domain scores at the final induction period visit found a statistically significant effect of remission status for all SIBDQ, SF-12v2, and WPAI:UC domains. As shown in Table 4, patients who achieved complete or partial remission following induction treatment scored significantly better on all SIBDQ, SF-12v2, and WPAI:UC domains than patients who were not in remission. Scores were comparable for patients in complete remission and in partial remission: mean differences were not statistically significant for any WPAI:UC domains, nor for 3 of 4 SIBDQ domains (all except Social Function) and for 7 of the 8 SF-12v2 domains (all except Bodily Pain).

**Table 4.** Effectiveness of Induction Treatment on Domains of HRQL and WRO as a Function of Remission Status: Comparison of Mean Scores at the Final Induction Period Visit for the Induction Sample

Measure	Not in Remission		Partial Remission		Complete Remission		Pairwise comparisons (Bonferroni-adjusted *P*-values)		
	N	LS Mean (SE)^a^	N	LS Mean (SE)^a^	N	LS Mean (SE)^a^	Partial vs. Not in Remission	Complete vs. Not in Remission	Partial vs. Complete Remission
**SIBDQ**									
Bowel Symptoms	155	14.0 (0.24)	186	17.8 (0.22)	124	18.4 (0.27)	d	d	*ns*
Systemic Symptoms	156	9.6 (0.18)	197	11.3 (0.16)	128	11.6 (0.20)	d	d	*ns*
Emotional Function	155	13.4 (0.26)	192	16.8 (0.23)	129	17.6 (0.28)	d	d	*ns*
Social Function	157	9.5 (0.19)	196	12.2 (0.17)	131	12.9 (0.21)	d	d	^b^
**SF-12v2**									
Physical Functioning	167	47.1 (0.61)	236	52.1 (0.52)	148	53.2 (0.65)	d	d	*ns*
Role Physical	164	44.9 (0.52)	234	51.0 (0.44)	147	52.2 (0.55)	d	d	*ns*
Bodily Pain	163	45.9 (0.57)	231	53.2 (0.48)	146	55.4 (0.60)	d	d	^b^
General Health	167	43.1 (0.64)	235	50.5 (0.54)	148	52.1 (0.68)	d	d	*ns*
Vitality	160	48.0 (0.70)	232	54.6 (0.58)	148	56.2 (0.73)	d	d	*ns*
Social Functioning	166	43.7 (0.61)	236	50.7 (0.51)	148	52.7 (0.64)	d	d	*ns*
Role Emotional	166	42.0 (0.63)	235	49.2 (0.54)	147	50.5 (0.68)	d	d	*ns*
Mental Health	166	44.8 (0.67)	236	53.1 (0.56)	148	53.2 (0.71)	d	d	*ns*
**WPAI:UC**									
Absenteeism	75	9.1 (1.68)	93	3.1 (1.52)	64	1.9 (1.82)	_b_	_b_	*ns*
Presenteeism	79	30.3 (2.05)	96	11.3 (1.87)	63	7.8 (2.27)	d	d	*ns*
Overall Work Impairment	74	35.6 (2.39)	92	13.5 (2.16)	61	9.1 (2.62)	d	d	*ns*
Activity Impairment	138	36.0 (1.65)	176	12.8 (1.46)	120	10.2 (1.76)	d	d	*ns*

HRQL, health-related quality of life; LS, least-squares; ns, not statistically significant (Bonferroni-adjusted *P* > 0.05); SE, standard error of the mean; SF-12v2, SF-12v2 Health Survey; SIBDQ, Short Inflammatory Bowel Disease Questionnaire; WPAI:UC, Work Productivity and Activity Impairment–Ulcerative Colitis.

Omnibus tests for a main effect of remission status were statistically significant for all models (all Hommel-adjusted *P* < 0.02).

a Derived from univariate one-way analysis of covariance models with remission status at the final induction period visit as a between-subjects factor, and with age, gender, disease duration, disease extent, and baseline value as covariates.

b Bonferroni-adjusted *P* < 0.05.

^c^Bonferroni-adjusted *P* < 0.01.

^d^Bonferroni-adjusted *P* < 0.001.

The percentages of induction sample patients in each remission status group who achieved clinically meaningful improvement (ie, change > MCID threshold) on SIBDQ total, SF-12v2 PCS and MCS, and WPAI:UC Overall Work Impairment scores following induction treatment are reported in Table 5. The proportion of patients showing meaningful improvements in each of these outcomes differed significantly as a function of remission status. Pairwise differences between each group found that patients who achieved either complete remission or partial remission were significantly more likely to show clinically meaningful improvement on each of these outcomes than patients who did not achieve remission. The proportion of patients in complete or partial remission showing meaningful change were comparable for these outcomes, with the exception of SF-12v2 PCS scores, for which patients in complete remission were more likely to demonstrate meaningful improvement.

**Table 5.** Proportion of Treatment Responders on HRQL and WRO in the Induction Sample Across Remission Status Groups at the Final Induction Period Visit

				Difference in Standardized Residuals		
Measure	Not in Remission	Partial Remission	Complete Remission	Partial vs. Not in Remission	Complete vs. Not in Remission	Partial vs. Complete Remission
SIBDQ Total	36.4	63.9	74.6	4.5^c^	5.9^c^	1.4
SF-12v2 PCS	30.1	49.8	63.5	3.8^c^	6.1^c^	2.3^a^
SF-12v2 MCS	30.1	55.7	58.8	5.0^c^	5.2^c^	0.2
WPAI:UC Overall Work Impairment	54.1	78.3	88.5	2.5^a^	3.3^b^	0.8

HRQL, health-related quality of life; MCID, minimal clinically important difference; MCS, Mental Component Summary; PCS, Physical Component Summary; SF-12v2, SF-12v2 Health Survey; SIBDQ, Short Inflammatory Bowel Disease Questionnaire; WPAI:UC, Work Productivity and Activity Impairment–Ulcerative Colitis; WRO, work-related outcomes.

Responders for each outcome were defined as showing improvement from baseline to the final induction period visit that exceeded the MCID threshold.

Omnibus chi-square tests were statistically significant for all models.

a Hommel-adjusted *P* < 0.05.

b Hommel-adjusted *P* < 0.01.

c Hommel-adjusted *P* < 0.001.

At the final maintenance period visit, statistically significant effects of remission status were observed for all SIBDQ, SF-12v2, and WPAI:UC domains. As shown in Table 6, patients in either complete remission or partial remission scored significantly better on all domains than patients not in remission. No differences in scores were observed between patients in complete or partial remission. SF-12v2 scores for patients in partial remission were at or above 50 on all domains, indicating that maintaining partial remission was sufficient for reaching normalized levels of functioning and well-being.

**Table 6.** Effectiveness of Maintenance Treatment on Domains of HRQL and WRO as a Function of Remission Status: Comparison of Mean Scores at the Final Maintenance Period Visit for the Maintenance Subset

Measure	Not in Remission		Partial Remission		Complete Remission		Pairwise Comparisons (Bonferroni-adjusted *P*-values)		
	N	LS Mean (SE)^a^	N	LS Mean (SE)^a^	N	LS Mean (SE)^a^	Partial vs. Not in Remission	Complete vs. Not in Remission	Partial vs. Complete Remission
**SIBDQ**									
Bowel Symptoms	85	13.8 (0.32)	72	18.2 (0.35)	103	18.8 (0.29)	d	d	*ns*
Systemic Symptoms	88	9.5 (0.22)	75	11.6 (0.24)	107	12.1 (0.20)	d	d	*ns*
Emotional Function	88	14.1 (0.31)	75	17.7 (0.33)	106	18.0 (0.28)	d	d	*ns*
Social Function	88	9.7 (0.22)	76	12.8 (0.24)	108	13.3 (0.20)	d	d	*ns*
**SF-12v2**									
Physical Functioning	100	47.7 (0.71)	87	54.0 (0.76)	117	54.0 (0.66)	d	d	*ns*
Role Physical	98	45.7 (0.67)	87	52.8 (0.72)	117	53.2 (0.62)	d	d	*ns*
Bodily Pain	98	46.4 (0.74)	87	54.1 (0.79)	115	55.0 (0.69)	d	d	*ns*
General Health	100	41.3 (0.81)	87	53.0 (0.87)	117	55.0 (0.75)	d	d	*ns*
Vitality	100	49.1 (0.89)	86	57.2 (0.97)	113	58.0 (0.85)	d	d	*ns*
Social Functioning	100	44.7 (0.78)	87	51.8 (0.84)	117	52.9 (0.72)	d	d	*ns*
Role Emotional	100	44.7 (0.73)	87	51.0 (0.79)	115	51.1 (0.68)	d	d	*ns*
Mental Health	100	47.6 (0.82)	87	54.4 (0.88)	117	55.5 (0.76)	d	d	*ns*
**WPAI:UC**									
Absenteeism	41	11.8 (1.95)	37	0.8 (2.07)	45	0.8 (1.85)	d	d	*ns*
Presenteeism	46	28.8 (2.44)	34	8.8 (2.83)	50	3.7 (2.32)	d	d	*ns*
Overall Work Impairment	40	38.4 (2.98)	34	9.0 (3.24)	44	4.8 (2.84)	d	d	*ns*
Activity Impairment	83	34.3 (1.98)	71	10.3 (2.15)	97	7.2 (1.84)	d	d	*ns*

HRQL, health-related quality of life; LS, least-squares; ns, not statistically significant (Bonferroni-adjusted *P* > 0.05); SE, standard error of the mean; SF-12v2, SF-12v2 Health Survey; SIBDQ, Short Inflammatory Bowel Disease Questionnaire; WPAI:UC, Work Productivity and Activity Impairment–Ulcerative Colitis; WRO, work-related outcomes.

Omnibus tests for a main effect of remission status were statistically significant for all models (all Hommel-adjusted *P* < 0.001).

a Derived from univariate one-way analysis of covariance models with remission status at the final maintenance period visit as a between-subjects factor, and with age, gender, disease duration, disease extent, and month 0 value as covariates.

^b^Bonferroni-adjusted *P* < 0.05.

^c^Bonferroni-adjusted *P* < 0.01.

^d^Bonferroni-adjusted *P* < 0.001.

**FIGURE 2.**
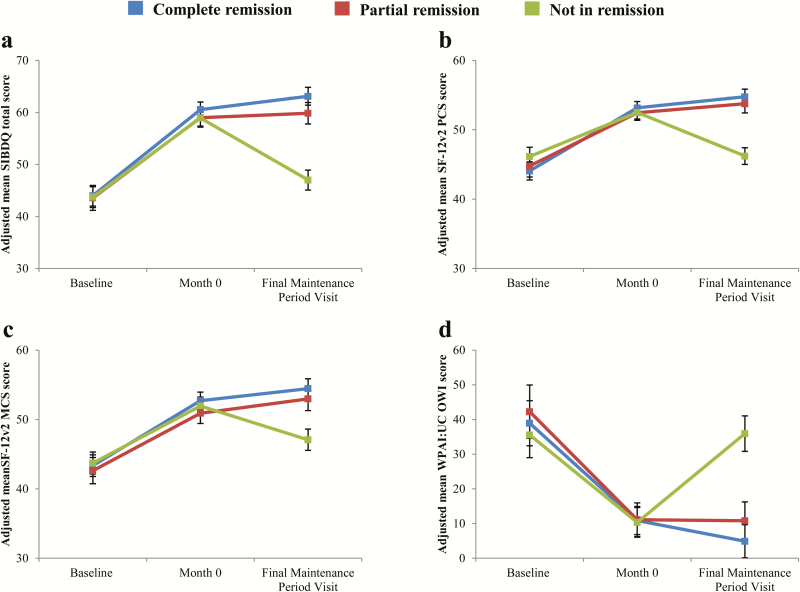
Changes in HRQL and WRO Across Induction and Maintenance Period Visits for the Maintenance Subset as a Function of Remission Status at the Final Maintenance Period Visit. Panel A: SIBDQ total score, Panel B: SF-12v2 PCS score, Panel C: SF-12v2 MCS score, Panel D: WPAI:UC overall work impairment (OWI) score. Adjusted means derived using mixed-effects models with repeated measures (MMRM) with patients’ age, gender, duration of disease, and disease extent at study baseline included as covariates. HRQL, health-related quality of life; MCS, Mental Component Summary; PCS, Physical Component Summary; SF-12v2, SF-12v2 Health Survey; SIBDQ, Short Inflammatory Bowel Disease Questionnaire; WPAI:UC, Work Productivity and Activity Impairment–Ulcerative Colitis; WRO, work-related outcomes.

Least-squares mean SIBDQ total, SF-12v2 PCS and MCS, and WPAI:UC Overall Work Impairment scores at baseline, week 8/month 0, and final maintenance period visits by remission status for patients in the maintenance subset are presented in Figure 2. Although all patient subgroups showed similar improvements during the induction phase, different patterns of change as a function of remission status were observed in the maintenance phase. For all 4 scores (Fig. 2A–D), patients not in remission at the final maintenance period visit showed a statistically significant decrease from month 0 to the final visit (all Bonferroni-adjusted *P*s < 0.001), while patients in partial remission and in complete remission showed either no change or continued improvement. The magnitude of deterioration in SF-12v2 PCS and WPAI:UC Overall Work Impairment scores for those not in remission in the maintenance phase indicated a complete reversion: their scores at the end of the maintenance period did not statistically differ from their scores at study baseline.

Results from models of changes in SIBDQ, SF-12v2, and WPAI:UC domain scores from month 0 to the final maintenance period visit as a function of remission status trajectory are presented in Table 7. For all domains, no statistically significant interaction effects nor main effects due to patients’ remission status at either visit were observed, indicating no differences in the magnitude of change in scores regardless of patients’ remission status at either visit.

**Table 7.** Change in HRQL and WRO During the Maintenance Period as a Function of Remission Status at Both Month 0 and at the Final Maintenance Period Visit for the Patients in the Maintenance Subset Who Were in Either Complete or Partial Remission at Both Visits

Measure	Partial Remission at Month 0				Complete Remission at Month 0						
	Partial Remission at Month 12		Complete Remission at Month 12		Partial Remission at Month 12		Complete Remission at Month 12		Tests for Main and Interaction Effects (Hommel-adjusted *P*-values)^a^		
	N	Mean Change (SE)	N	Mean Change (SE)	N	Mean Change (SE)	N	Mean Change (SE)	Remission Status at Month 0	Remission Status at Month 12	Interaction
**SIBDQ**											
Bowel Symptoms	47	0.2 (0.31)	52	0.5 (0.29)	25	-0.1 (0.43)	51	0.8 (0.30)	*ns*	*ns*	*ns*
Systemic Symptoms	49	0.1 (0.23)	53	0.8 (0.22)	26	0.0 (0.33)	54	0.5 (0.23)	*ns*	*ns*	*ns*
Emotional Function	48	0.6 (0.28)	53	0.5 (0.27)	27	-0.3 (0.38)	53	0.7 (0.27)	*ns*	*ns*	*ns*
Social Function	49	0.1 (0.19)	53	0.8 (0.18)	27	0.3 (0.26)	55	0.6 (0.18)	*ns*	*ns*	*ns*
**SF-12v2**											
Physical Functioning	59	1.9 (0.70)	57	1.3 (0.70)	28	0.7 (1.02)	60	1.7 (0.69)	*ns*	*ns*	*ns*
Role Physical	59	1.4 (0.69)	57	1.5 (0.70)	28	0.6 (1.02)	60	1.8 (0.69)	*ns*	*ns*	*ns*
Bodily Pain	59	0.5 (0.69)	56	0.6 (0.70)	28	-0.6 (1.01)	59	1.5 (0.69)	*ns*	*ns*	*ns*
General Health	59	2.6 (0.81)	57	4.1 (0.82)	27	-0.1 (1.20)	59	4.0 (0.81)	*ns*	*ns*	*ns*
Vitality	59	3.3 (1.02)	54	2.7 (1.05)	28	-0.1 (1.52)	60	3.3 (1.02)	*ns*	*ns*	*ns*
Social Functioning	59	0.9 (0.87)	57	1.1 (0.87)	28	-0.8 (1.26)	60	1.6 (0.86)	*ns*	*ns*	*ns*
Role Emotional	59	1.7 (0.74)	57	1.8 (0.75)	28	1.6 (1.09)	58	1.7 (0.76)	*ns*	*ns*	*ns*
Mental Health	59	2.4 (0.87)	57	2.5 (0.88)	28	-0.8 (1.28)	60	2.3 (0.87)	*ns*	*ns*	*ns*
**WPAI:UC**											
Absenteeism	23	-1.3 (0.51)	21	-1.2 (0.53)	14	-1.3 (0.65)	24	-1.9 (0.49)	*ns*	*ns*	*ns*
Presenteeism	19	-1.6 (2.00)	26	-5.2 (1.70)	15	2.9 (2.25)	24	-4.4 (1.76)	*ns*	*ns*	*ns*
Overall Work Impairment	20	-4.1 (2.29)	21	-5.0 (2.23)	14	2.7 (2.75)	23	-6.2 (2.13)	*ns*	*ns*	*ns*
Activity Impairment	45	-0.4 (1.74)	44	-4.7 (1.76)	26	0.2 (2.32)	53	-1.9 (1.61)	*ns*	*ns*	*ns*

HRQL, health-related quality of life; ns, not statistically significant (Hommel-adjusted *P* > 0.05); SE, standard error of the mean; SF-12v2, SF-12v2 Health Survey; SIBDQ, Short Inflammatory Bowel Disease Questionnaire; WPAI:UC, Work Productivity and Activity Impairment–Ulcerative Colitis; WRO, work-related outcomes.

a Derived from univariate two-factor analysis of covariance (ANCOVA) models with remission status at month 0, remission status at month 12, and the remission status at month 0 × remission status at month 12 interaction as fixed factors, with age, gender, disease duration, disease extent, and month 0 value as covariates.

Statistically significant predictors of change in SIBDQ, SF-12v2, and WPAI:UC domain scores during the induction and maintenance periods from multivariable regression models are presented in Table 8. When accounting for the impact of patients’ age, gender, disease duration, and disease extent at study baseline, remission status at period endpoint and score at period baseline were statistically significant predictors of changes in all domains. Disease duration was a statistically significant predictor of 3 of 4 SF-12v2 mental-based domains (Vitality, Role Emotional, and Mental Health) during the induction period.

**TABLE 8.** Statistically Significant Predictors of Change in Domains of HRQL and WRO During the Induction and Maintenance Periods Derived Using Multivariable Regression Models

	Induction Period^a^				Maintenance Period^b^			
	Predictors^c^	*β*	*P*-value	Adjusted R^2^	Predictors^c^	*β*	*P*-value	Adjusted R^2^
**SIBDQ**								
Bowel	Remission status	0.43	f	0.46	Remission status	0.54	f	0.36
	Baseline score	-0.57	f		Baseline score	-0.34	f	
Systemic	Remission status	0.27	f	0.40	Remission status	0.43	f	0.34
	Baseline score	-0.60	f		Baseline score	-0.44	f	
Emotional	Remission status	0.38	f	0.43	Remission status	0.47	f	0.28
	Baseline score	-0.54	f		Baseline score	-0.31	f	
	Gender	0.09	e					
Social	Remission status	0.38	f	0.50	Remission status	0.53	f	0.39
	Baseline score	-0.61	f		Baseline score	-0.37	f	
**SF-12v2**								
Physical Functioning	Remission status	0.22	f	0.44	Remission status	0.30	f	0.32
	Baseline score	-0.62	f		Baseline score	-0.51	f	
					Age	-0.13	d	
Role Physical	Remission status	0.30	f	0.45	Remission status	0.37	f	0.32
	Baseline score	-0.63	f		Baseline score	-0.46	f	
Bodily Pain	Remission status	0.34	f	0.53	Remission status	0.40	f	0.29
	Baseline score	-0.64	f		Baseline score	-0.37	f	
General Health	Remission status	0.30	f	0.47	Remission status	0.53	f	0.39
	Baseline score	-0.60	f		Baseline score	-0.40	f	
Vitality	Remission status	0.27	f	0.43	Remission status	0.33	f	0.34
	Baseline score	-0.62	f		Baseline score	-0.52	f	
	Disease duration	-0.07	d					
Social Functioning	Remission status	0.32	f	0.47	Remission status	0.37	f	0.28
	Baseline score	-0.63	f		Baseline score	-0.42	f	
Role Emotional	Remission status	0.29	f	0.45	Remission status	0.31	f	0.27
	Baseline score	-0.59	f		Baseline score	-0.44	f	
	Disease duration	-0.08	d					
Mental Health	Remission status	0.28	f	0.41	Remission status	0.34	f	0.27
	Baseline score	-0.58	f		Baseline score	-0.44	f	
	Disease duration	-0.08	d					
**WPAI:UC**								
Absenteeism	Remission status	-0.12	e	0.61	Remission status	-0.27	f	0.42
	Baseline score	-0.78	f		Baseline score	-0.61	f	
Presenteeism	Remission status	-0.33	f	0.53	Remission status	-0.52	f	0.36
	Baseline score	-0.68	f		Baseline score	-0.35	f	
Overall Work Impairment	Remission status	-0.35	f	0.51	Remission status	-0.55	f	0.43
	Baseline score	-0.65	f		Baseline score	-0.38	f	
Activity Impairment	Remission status	-0.36	f	0.52	Remission status	-0.49	f	0.35
	Baseline score	-0.66	f		Baseline score	-0.36	f	
					Age	0.12	d	

HRQL, health-related quality of life; SF-12v2, SF-12v2 Health Survey; SIBDQ, Short Inflammatory Bowel Disease Questionnaire; WPAI:UC, Work Productivity and Activity Impairment–Ulcerative Colitis; WRO, work-related outcomes.

a Derived from multivariable linear regression models with change in value from baseline to final induction visit as the outcome variable, and with predictors including remission status at the final induction period visit, age, gender, disease duration, disease extent, and baseline value.

b Derived from multivariable linear regression models with change in value from month 0 to final maintenance visit as the outcome variable, and with predictors including remission status at the final maintenance period visit, age, gender, disease duration, disease extent, and month 0 value.

c Only predictors with statistically significant regression weights (ie, *P* < 0.05) are included in the table.

^d^ *P* < 0.05.

^e^ *P* < 0.01.

^f^ *P* < 0.001.

## DISCUSSION

The primary objective of the current analyses was to examine whether HRQL and WRO of adult patients with mild-to-moderate UC who achieved or maintained partial remission following treatment with multimatrix mesalamine would more closely match those of patients who achieved or maintained complete remission or those of patients who were not in remission following treatment. The findings observed from this study support the former.

Patients with active UC who achieved partial remission after completing 8 weeks of induction treatment scored significantly better on all measured aspects of HRQL and WRO than did patients who did not complete treatment or did not achieve remission during this period. Further, patients in partial remission after 12 months of maintenance treatment scored significantly better on all HRQL and WRO domains than patients who did not complete treatment or did not achieve remission during this period. At the same time, patients in partial remission following induction treatment were not statistically distinguishable from those who achieved complete remission on any domains of the WPAI:UC, nor on any SIBDQ domains with the exception of Social Function and any SF-12v2 domains with the exception of Bodily Pain, whereas partial and complete remission subgroups did not statistically differ on any of these domains after 12 months of maintenance treatment. Additionally, when examining changes in outcomes during the maintenance treatment, patients who maintained complete or partial remission at the end of the maintenance phase showed stable or even continued improvement in HRQL and WRO, whereas patients who did not maintain remission showed significant deterioration in all outcomes. Further, fluctuations between complete and partial remission during the maintenance period had no impact on any HRQL or WRO domains. Finally, changes in all domains during each treatment period were significantly predicted by remission status when controlling for patients’ age, gender, disease duration, and disease extent.

These results provide evidence that, for the humanistic outcomes evaluated here, the benefits of achieving partial remission were not different from those associated with complete remission. Certainly, the primary goal of UC treatment should be producing full and complete remission. However, restoring patients’ functional health and well-being, and removing impairments to their ability to work productively and engage in other activities, are achievable goals even if complete remission of the disease has not been reached.

Although patients in the current study were treated with multimatrix mesalamine, we do not make the assumption that our findings regarding the impact of remission status on HRQL and WRO are specific to this particular drug, nor to any particular regimen; that is, we believe that these effects are treatment- agnostic. We would thus speculate that these findings would be replicated in a UC sample receiving any effective UC treatment that induced partial or complete remission. Our analyses found that patients in complete or partial remission who relapsed at the end of the maintenance phase despite continued treatment not only showed significant declines in their HRQL and WRO, but for many outcomes there was a complete regression to their pretreatment levels. We would expect to observe the same patterns for patients in complete or partial remission who discontinued treatment. These speculations derive from our general assumption that treatment itself is not the direct or proximal agent of improvements in HRQL and WRO, but rather its effect is indirect, mediated through remission status. Future UC treatment studies using randomized controlled designs would be able to test these hypotheses.

Another important consideration of these results is that even substantial improvements in patients’ HRQL and WRO (including normalized levels of functioning and well-being) do not necessarily signify that complete clinical remission has been achieved. Practicing clinicians often rely upon patient’s reports about perceived improvements in daily functioning and well-being as an indicator of treatment success,^1^ and so could erroneously assume that full remission has been achieved in cases for which these improvements are in fact a product of only partial remission. Patients themselves are likely to make similar assumptions based on improvements in these secondary indicators, underestimating their disease activity, which could lead to a purposeful reduction in treatment adherence and result in disease relapse.^36,37^ Thus, both clinicians and patients alike should interpret HRQL and WRO benefits with some caution, since important clinical gains should still be pursued for optimal management of the disease.

Relatedly, while achievement of complete remission in this trial required the absence or reduction in both clinical and endoscopic signs, achievement of partial remission required improvements only in clinical signs.

The current findings thus indicate that mucosal healing might not be a necessary condition for significant improvements in HRQL and WRO, and consequently that observed improvements in HRQL and WRO should not be inferred as evidence of mucosal healing. Historically, most clinical trials have defined remission only with respect to clinical symptoms, particularly evidence for rectal bleeding and increased stool frequency. Although some researchers have found data indicating that evaluating endoscopic signs does not add significant diagnostic information to noninvasive measures of clinical activity alone,^26,38–40^ other evidence suggests that measures of clinical disease activity are in fact poor predicators of underlying mucosal health, with an estimated one-third to one-half of patients with UC in clinical remission who suffer from mucosal inflammation.^41–44^ The numerous benefits of mucosal healing, such as predicting reduced risks of clinical relapse,^45,46^ need for future colectomy,^47–49^ and development of colorectal cancer^50,51^ support the inclusion of endoscopic disease activity as an endpoint in clinical trials of UC, regardless of whether this activity shows immediate impact on patient-reported HRQL and WRO.

Several limitations of this study and analysis constrain the interpretation of our findings. One key limitation is that, although this open-label, nonblinded trial may closely match the manner in which UC treatment is used in real-world settings, the lack of a randomized controlled design limits the ability to determine the causal relations between treatment, remission status, and HRQL and WRO. Whereas remission status was strongly predictive of these outcomes, these data cannot definitively answer if remission status was a direct or even indirect cause of patients’ HRQL or WRO. Further, we also are hindered from making causal inferences regarding the role of multimatrix mesalamine treatment in directly (or indirectly) producing disease remission or improvements in HRQL and WRO.

Another issue for consideration is whether SF-12v2 norm-based scores, which are calculated using algorithms derived from a US general population sample, can be appropriately interpreted when the instrument is used in non-US samples. Results from the International Quality of Life Assessment (IQOLA) project,^52^ which had the objective of validating and norming the SF-36 (from which the SF-12 is directly derived) in a large international study, found equivalence in scores calculated using the US-based algorithm with those calculated from country-based algorithms across 9 non-US countries.^53^ However, some researchers have found differences in SF-36 or SF-12 scores when applying US-based and country-specific algorithms and advocate for the use of country-specific norm-based scoring.^54–56^ Further, because only Western European countries were included in the IQOLA comparison, it is thus possible that the generalizability of the US-based algorithms extended only to countries that share similar cultural and economic structures with the US. Given that citizens’ actual and perceived health status are strongly associated with their country’s cultural and socioeconomic status,^57^ it may be the case that the US-based algorithms are inappropriate when used with patients in several of the countries included in the current study, such as Eastern Europe nations (eg, Poland, Romania, and Czech Republic) and those in Africa (South Africa), Asia (India), and South America (Colombia). To examine the influence of country on our results, we conducted an exploratory post hoc sensitivity analysis for which we respecified all ANCOVA models to include patients’ country as a categorical factor. Findings from this analysis indicated that, although country had a small but significant association with several HRQL and WRO domains, there was virtually no impact in the effects of remission status on these outcomes from the original models [results not shown]. Still, future clinical studies administering the SF-12 or SF-36 at international sites should consider the implications for using standard or country-specific scoring in order to maximize interpretation of their results.

Another limitation is that due to both administrative causes as well as the lack of validated linguistic translations of the SIBDQ and WPAI:UC for some of the countries included in this trial, PRO surveys were not made available to all trial patients at all visits. Administration rates for surveys at baseline were 77% for the SIBDQ, 87% for the SF-12v2, and 45% for the WPAI:UC. Examination of investigator reports and patterns of missing data identified 10 sites in 2 countries—Colombia (all 4 sites) and India (6 of 14 sites)—where PRO instruments were not administered in a manner consistent with the protocol. Because these data were not missing at random, but rather were linked to countries and sites, the possibility that associations of country- or site-related variables with treatment effectiveness, remission status, HRQL, and WRO may have produced systematic bias in our results. To examine the degree to which this was the case, we conducted an exploratory post hoc sensitivity analysis to test all inferential models for all outcomes when excluding patients from these 10 sites from the analysis sample. Findings from the sensitivity analysis [results not shown] were essentially equivalent with findings from original models, indicating that the inclusion of patients from these sites did not meaningfully impact our interpretations of and overall conclusions from these data. Still, the impact of these protocol violations cannot be completely accounted for, and the existence of resulting bias cannot be definitively ruled out. It also should be noted that because of these administrative issues, the size of the analysis sample was smaller than anticipated, which could have resulted in inflated type II error due to insufficient statistical power.

In conclusion, adults with moderate-to-severe UC who achieved partial remission following short-term induction treatment with 4.8 g/day multimatrix mesalamine showed improvements in HRQL and WRO that were of equivalent magnitude to those of patients who achieved complete remission. Further, patients in partial and complete remission groups also had equivalent HRQL and WRO following 12 months of maintenance treatment with 2.4 g/day multimatrix mesalamine. These findings suggest that even partial clinical remission leads to meaningful improvements in patients’ self-rated functioning, well-being, and work performance.

## Notes

Conflict of Interest: Mary Kaye Willian and Megan Teynor were employees of Shire at the time of the study, analysis, and/or development of this manuscript and were holding stock and/or stock options at Shire. Geert D’Haens has received financial support for research from AbbVie, Janssen Biologics, Given Imaging, MSD; Dr Falk Pharma, and Photopill; received lecture fees from AbbVie, Tillotts, Tramedico, Ferring, MSD, UCB, Norgine, and Shire; and consulted for AbbVie, Actogenix, Centocor, Cosmo, Engene, Ferring Pharmaceuticals, GlaxoSmithKline, Janssen Biologics, Millennium Pharmaceuticals, MSD, Novo Nordisk, PDL Biopharma, Pfizer, SetPoint, Shire, Takeda, Teva, and UCB. Aaron Yarlas is an employee of Optum, which received funds from Shire Development LLC for data analyses.

Supported by: Funding for this research was provided by Shire Development LLC.

## Abbreviations

ANCOVA: Univariate one-way Analysis of Covariance
ANOVA: Analysis of Variance
BP: Bodily Pain
GH: General Health
HRQL: Health-Related Quality of Life
IBD: Inflammatory Bowel Disease
IBDQ: Inflammatory Bowel Disease Questionnaire
MCID: Minimal Clinically Important Difference
MCS: Mental Component Summary
MH: Mental Health
MMRM: Mixed-effects Models with Repeated Measures
_MOD_UC-DAI: Modified Version of the Ulcerative Colitis Disease Activity Index
PCS: Physical Component Summary
PF: Physical Functioning
PGA: Physician Global Assessment
PRO: Patient-Reported Outcomes
QD: Once Daily
RE: Role limitations due to Emotional health problems
RP: Role limitations due to Physical health problems
SD: Standard Deviation
SF: Social Functioning
SF-12v2: SF-12v2 Health Survey
SF-36v2: SF-36v2 Health Survey
SIBDQ: Short Inflammatory Bowel Disease Questionnaire
UC: Ulcerative Colitis
UC-DAI: Ulcerative Colitis Disease Activity Index
US: United States
VT: Vitality
WPAI: Work Productivity and Activity Impairment
WPAI:UC: Work Productivity and Activity Impairment—Ulcerative Colitis
WRO: Work-Related Outcomes.
